# Running Speed Estimation Using Shoe-Worn Inertial Sensors: Direct Integration, Linear, and Personalized Model

**DOI:** 10.3389/fspor.2021.585809

**Published:** 2021-03-18

**Authors:** Mathieu Falbriard, Abolfazl Soltani, Kamiar Aminian

**Affiliations:** Laboratory of Movement Analysis and Measurement, École Polytechnique Fédérale de Lausanne, Lausanne, Switzerland

**Keywords:** IMUs, speed, running, overground, linear prediction, personalization

## Abstract

The overground speed is a key component of running analysis. Today, most speed estimation wearable systems are based on GNSS technology. However, these devices can suffer from sparse communication with the satellites and have a high-power consumption. In this study, we propose three different approaches to estimate the overground speed in running based on foot-worn inertial sensors and compare the results against a reference GNSS system. First, a method is proposed by direct strapdown integration of the foot acceleration. Second, a feature-based linear model and finally a personalized online-model based on the recursive least squares' method were devised. We also evaluated the performance differences between two sets of features; one automatically selected set (i.e., optimized) and a set of features based on the existing literature. The data set of this study was recorded in a real-world setting, with 33 healthy individuals running at low, preferred, and high speed. The direct estimation of the running speed achieved an inter-subject mean ± STD accuracy of 0.08 ± 0.1 m/s and a precision of 0.16 ± 0.04 m/s. In comparison, the best feature-based linear model achieved 0.00 ± 0.11 m/s accuracy and 0.11 ± 0.05 m/s precision, while the personalized model obtained a 0.00 ± 0.01 m/s accuracy and 0.09 ± 0.06 m/s precision. The results of this study suggest that (1) the direct estimation of the velocity of the foot are biased, and the error is affected by the overground velocity and the slope; (2) the main limitation of a general linear model is the relatively high inter-subject variance of the bias, which reflects the intrinsic differences in gait patterns among individuals; (3) this inter-subject variance can be nulled using a personalized model.

## Introduction

The overground speed is the most useful metric in training and performance analysis of running. Researchers have tried for decades to understand the physiological and biomechanical adjustments occurring at different ranges of running speeds (Williams and Cavanagh, 1987; Nummela et al., 2007; Moore, 2016; Thompson, 2017). However, most of the existing studies were performed in a controlled environment (i.e., treadmill running inside a laboratory) where the runner has to adapt his gait to run at a constant speed. In overground running, change of environment, surface, slope, obstacles, and turns alter the gait and the running speed. Many studies have discussed the biomechanical adaptations associated with running on a treadmill vs. running overground (Van Hooren et al., 2019). While standard motion capture (i.e., stereophotogrammetry and force plate) offers accurate measurements in laboratories, the recent emergence of wearable systems is paving the shift toward studies carried overground and in real-world conditions (Benson et al., 2018).

The real-world estimation of the overground speed is generally obtained using a body-worn Global Navigation Satellite System (GNSS). Although these systems provide accurate and reliable measurement of the locomotion speed (Terrier et al., 2000; Witte and Wilson, 2004), they suffer from several limitations: (1) their high power consumption restricts their duration of use in portable devices, (2) the communication between the receiver and the satellite is not always guaranteed (e.g., indoor, near high buildings), and (3) the measurement accuracy decrease during rapid changes of speed and position (Varley et al., 2012; Rawstorn et al., 2014). As a solution to the latter limitation, systems based on the data fusion of body-worn inertial and GNSS sensors have been proposed to monitor sports activities (Brodie et al., 2008; Waegli and Skaloud, 2009; Zihajehzadeh et al., 2015). However, to address the issue of power consumption and communication losses, IMU-based systems must be able to estimate the speed without or with very limited input from a GNSS device.

Several methods have been proposed to estimate the walking speed using IMUs attached to different body-segments (Miyazaki, 1997; Aminian et al., 2002; Zijlstra and Hof, 2003; Sabatini et al., 2005; Hu et al., 2013; Salarian et al., 2013). One solution would be to extend and adapt these methods to running. However, these methods often relied on walking models or on the estimation of step length, which cannot be directly applied to running because of the aerial phases, where accelerometers are erroneous. Other studies have used machine learning techniques to estimate the walking speed but did not validate the results for running (Zihajehzadeh and Park, 2016; Fasel et al., 2017).

To the authors' knowledge, few studies proposed an accurate ambulatory method, based on body-worn IMUs, to estimate the overground speed of running, and even less did so for instantaneous speed estimation. Two studies used a similar method (integration of the acceleration signal) to calculate the velocity of the shank (Yang et al., 2011) and foot (Chew et al., 2017) segments. However, the error of the system was computed over multiple strides, in a small range of speeds, and for level treadmill running. As mentioned previously, the velocity estimated from the integration of segment acceleration has limitations, particularly when the flight phase varies in a wide range or when various slopes are experienced as it is the case in overground running. Another study (Hausswirth et al., 2009) compared in-lab a commercialized speed estimation device with the speed of a treadmill and reported a relatively low accuracy considering that the system required a subject-specific calibration. Subject-specific neural networks were also devised to assess the running speed in free-living conditions using only triaxial accelerometric measurements, but the model needed a calibration/learning phase for each runner and was validated for the mean speed using few trials (Herren et al., 1999). One study, however, exploited the personalized calibration and proposed a model based solely on the contact time (De Ruiter et al., 2016). Although the authors obtained a low root-mean-square error (<3%), these results were not instantaneous estimations but rather the average speed over bouts of 125 meters. Besides, a more recent study (Soltani et al., 2019) based on wrist-worn inertial sensors suggested that better results could be achieved by including more features to the model.

The objective of the current study was three-fold: first, we aimed to extend an existing walking algorithm based on strapdown integration of foot acceleration and show its limitation for running speed estimation. Then we proposed a new linear model to predict the running speed at each step and in real-world condition, based on relevant features extracted from feet acceleration and angular velocity. Finally, we investigated how personalization improved the performances of the system using additional data, such as occasional GNSS inputs. We compared each method to the GNSS speed, considered as the ground truth, obtained during outdoor measurements of overground running, at different speeds and slopes.

## Materials and Methods

### Protocol and Instrumentation

**Figure 1:**
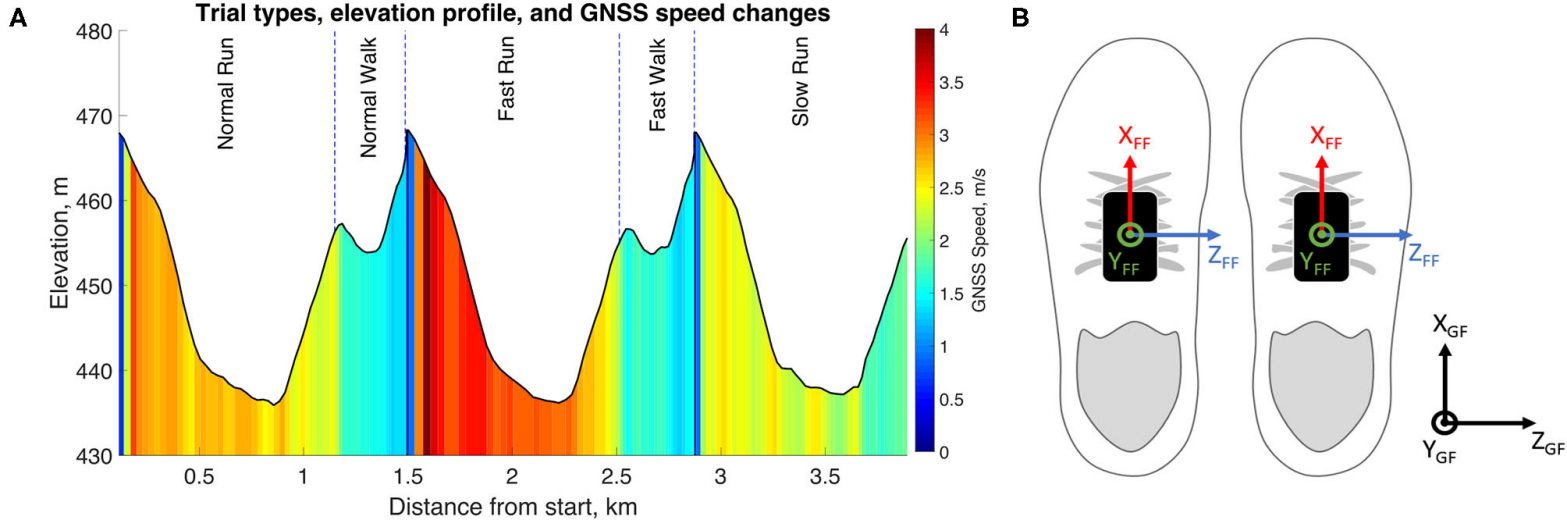
**(A)** The elevation and speed of the running circuit. This figure was adapted from Soltani et al. (2019). **(B)** The definition of the foot functional frame (FF) and global frame (GF) used in this study.

Thirty-three healthy and active participants [18 males (age: 38 ± 9 y.o.; size: 180 ± 7 cm; weight: 76 ± 9 kg), 15 females (age: 36 ± 10 y.o.; size: 165 ± 7 cm; weight: 59 ± 7 kg)] without any symptomatic musculoskeletal injuries participated to this study. The measurements were performed in real-world conditions with sections of uphill, downhill, and level running. We asked the participants to run the same circuit three times, once at self-adjusted normal, fast, and slow speeds (Figure 1A). The periods of rest and the walking bouts, in between the running segments, were manually removed from the analysis. The local ethics committee approved the present protocol, and we conducted the measurements in agreement with the declaration of Helsinki.

Each participant was equipped with two time-synchronized sensors (Physilog 4, Gait Up, Switzerland) strapped on the dorsum of the shoe. Each sensor included a triaxial accelerometer, a triaxial gyroscope, and a barometer. The barometer was sampled at 50 Hz. Acceleration (±16 g) and angular velocity (±2,000 deg/s) were recorded at 500 Hz and were calibrated according to Ferraris et al. (1955) before each measurement session. Furthermore, a GNSS receiver (CAMM8Q, u-blox, CH) with an external active antenna (ANN-MS, u-blox, CH) was mounted on the head using Velcro attached to a cap. GNSS was used as a reference system for the estimation of the running speed. The GNSS receiver was set to pedestrian mode with a sampling frequency of 10 Hz. With such a configuration, the datasheet of the manufacturer reported a median error of 0.05 m/s for instantaneous speed estimation. MATLAB software (R2018b, MathWorks, Natick, MA USA) was used for all the data processing steps without the need for publicly available libraries.

### Estimation of Reference GNSS Speed

**Figure 2:**
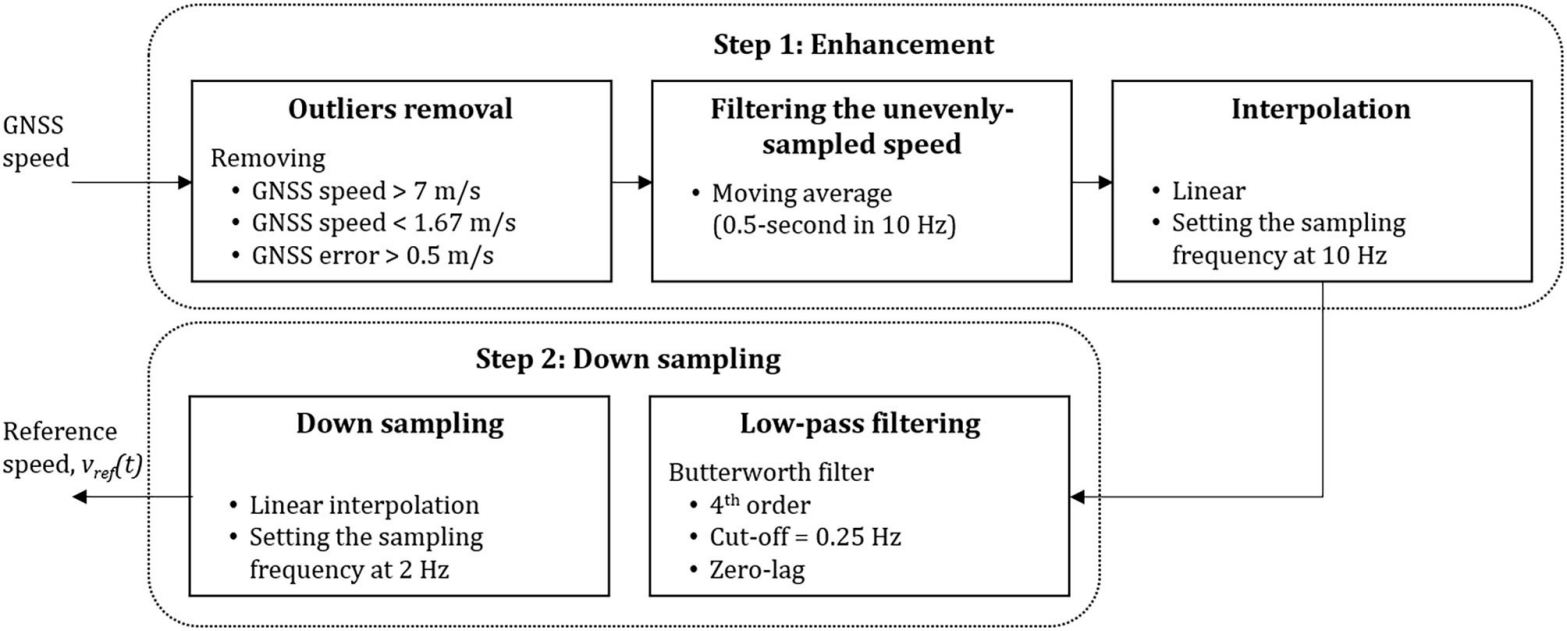
Pre-processing steps applied to the GNNS measurements of speed to obtain the reference speed estimation. This figure was adapted from Soltani et al. (2019).

The reference speed obtained from the GNSS receiver was processed according to Soltani et al. (2019) and in two steps (Figure 2). First, we enhanced the signal by removing the outliers that did not correspond to running; hence, we removed all recorded speed samples outside of the 5–20 km/h range. Moreover, the GNSS receiver provided an estimation of the accuracy of each observation; hence we discarded any data-point with an error higher than 0.15 m/s. This process retrieved an unevenly sampled reference speed signal. We applied a moving average of 0.5-s width (in 10 Hz), followed by linear interpolation to obtain an equally-spaced time series at 10 Hz. In the second step, the signal was down-sampled to provide the reference speed (v_ref_), after a fourth-order low-pass Butterworth filter with the cut-off frequency at 0.25 Hz to reduce the noise.

### Speed Estimation Based on Direct Integration of Foot Acceleration

**Figure 3:**
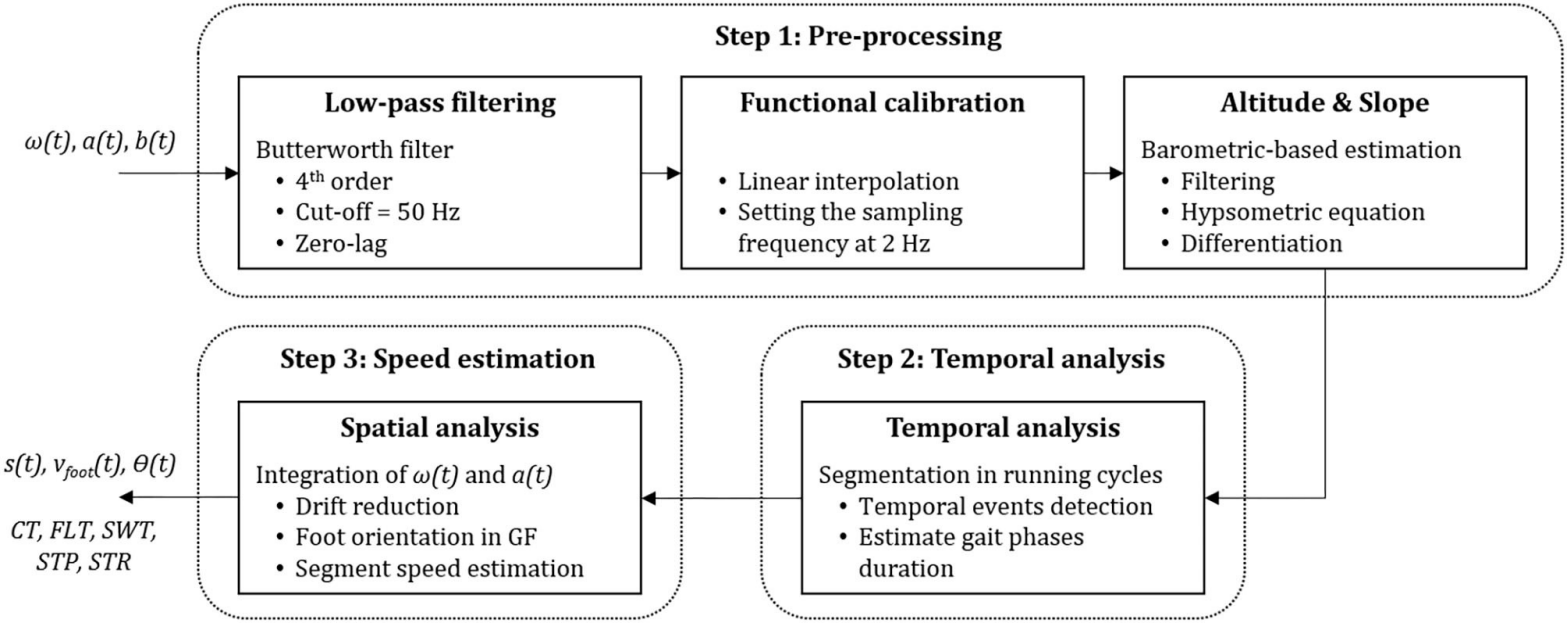
Steps performed on the IMU acceleration *a(t)*, angular velocity ω*(t)*, and barometric pressure *b(t)* measurements. The outputs were later used for feature extraction; the slope *s(t)*, the speed of the foot *v*_*foot*_*(t)*, the pitch angle θ*(t)*, contact time *CT*, flight time *FLT*, swing phase duration *SWT*, step duration *STP* and stride duration *STR*.

In this section, we describe the sequence of transformations that we applied on the IMU and barometer data to extract the gait features. The whole process can be summarized in four tasks: pre-processing, temporal analysis, spatial analysis, and foot speed estimation (Figure 3).

#### Pre-processing

First, a 4th-order low-pass Butterworth filter (Fc = 50 Hz) was applied on the raw acceleration (*a(t)*) and angular velocity (ω*(t)*) signals to reduce the noise. Then the IMU signals were aligned with the foot segment by computing the rotation matrix that transforms the data recorded in the technical frame of the sensors into the functional frame (FF) of the foot (Figure 1B). For this purpose, we used the measurements of level normal walking (Figure 1A) and a previously reported calibration method (Falbriard et al., 2018). This process aligned the y-axis of the IMU with the vertical axis of the foot, pointing upward, the z-axis to the mediolateral axis, pointing to the right side of the subject, and the x-axis to the longitudinal axis, pointing toward the forefoot. Throughout this paper, if not mentioned otherwise, the data are reported in the functional frame of the foot.

The last phase in pre-processing was estimating the overground slope. As the mechanics of running differ between level, uphill, and downhill running (Vernillo et al., 2017), we assumed that the elevation difference between successive steps would be a relevant input for the model. Therefore, the barometric pressure was converted by the hypsometric equation to the altitude signal (Bolanakis, 2017) smoothed by applying a 4-s moving average filter and down-sampled to 1 Hz time-series. The slope (*s(t)*) was defined as the altitude difference between two samples spaced by 5 s, by assuming that changes of altitude shorter than 5 s would not have a significant effect on the running speed.

#### Temporal Analysis

Temporal events detection was performed as described in Falbriard et al. (2018) by segmenting the race into mid-swing to mid-swing cycles and detecting of several temporal events within each cycle. Mid-swings were detected as the positive peaks observed on the pitch axis (FF z-axis) of the angular velocity measurements. Moreover, we improved the robustness of the peak detection algorithm by applying the YIN auto-correlation method (De Cheveign and Kawahara, 2002) over a 10-s sliding window (5-s overlap) to obtain an approximation of the cadence and set an adequate minimum time difference between two peaks. The initial contact event (IC), defined as the moment when the foot initiates contact with the ground at landing, and terminal contact (TC), defined as the instant when the toes leave the ground during the pushing phase, were then detected within each cycle using the two minimums of the pitch angular velocity. Moreover, we defined the event MinRot as the time-point where the norm of the angular velocity (||ω*(t)*||) is minimum within the stance phase (i.e., between IC and TC).

#### Spatial Analysis and Foot Speed Estimation

This process aimed to measure the orientation of the foot in the global frame (GF), remove the Earth's gravitational acceleration from the recorded acceleration, and integrate the corrected acceleration to obtain the speed of the foot. In GF, the x-axis was in the running direction, the z-axis corresponds to the axis perpendicular to the ground surface, and the y-axis was defined by the cross-product of the z and x-axes (Figure 1B). Using a previously validated technique (Falbriard et al., 2020), foot orientation was obtained in GF, and foot acceleration in FF was expressed in GF and the gravitational acceleration (*g* = [0 0 9.81] m/s^2^) removed. The resulting acceleration (in GF) was integrated using a trapezoidal rule to get a first estimate of the speed of the foot. We considered the speed of the foot to be zero during the stance phase and, therefore, estimated and removed the integration drift by linearly resetting the speed between MinRot and TC of each stance phase. Note that we preferred MinRot to the IC for drift resting since MinRot corresponds to the time sample when the foot is the closest to a static state, reportedly used as the integration limits in walking gait analysis (Mariani et al., 2010). We finally applied the inverse of the quaternions mentioned above to get the drift-corrected speed of the foot segments (*v*_*foot*_*(t)*) in the FF.

### Development of a Linear Model for Speed Prediction

#### Feature Extraction, Linearization, and Outliers Removal

First, we extracted several parameters (p_j_) for each step, which were later used as inputs for the speed estimation model. As several studies reported on the association between the changes in the duration of the gait phases and the running speed (Högberg, 1952; Saito et al., 1974; Nummela et al., 2007), we computed the ground contact time (CT), the flight time (FLT), the swing time (SWT), the step duration (STP), and the stride duration (STR) for each step i, where i = 1…N, and N is the total number of steps (Equations 1–5).

(1) $$\begin{array}{r} {CT}_{i}={TC}_{i}-\;{IC}_{i} \end{array}$$

(2) $$\begin{array}{r} {FLT}_{i}={IC}_{i+1}-{TC}_{i} \end{array}$$

(3) $$\begin{array}{r} {SWT}_{i}={IC}_{i+2}-{TC}_{i} \end{array}$$

(4) $$\begin{array}{r} {STP}_{i}={IC}_{i+1}-\;{IC}_{i} \end{array}$$

(5) $$\begin{array}{r} {STR}_{i}={IC}_{i+2}-\;{IC}_{i} \end{array}$$

As a few strides suffered from misdetections, outliers were removed according to (1) a valid stride must last between 0.37 and 2.5 s, and (2) the flight phase (FLT) must be >0.

Pitch angle (θ) at the IC was extracted as the angle between the longitudinal axis of the foot (FF x-axis) and the ground surface (x and y-axis in GF). A positive pitch angle corresponds to a rear-foot landing (i.e., talus region lower than the toes) and a negative pitch angle to a forefoot strike.

We also extracted several statistics from the acceleration *a(t)*, the angular velocity ω*(t)*, the foot speed *v*_*foot*_*(t)*, and the slope *s(t)* time-series. Moreover, since *a(t)*, ω*(t)*, and *v*_*foot*_*(t)* were 3-dimensional signals, these statistics were computed for each axis (i.e., x, y, and z) and the norm of the signal. Note that the features were captured on the signals of a single stride (i.e., between IC_i_ and IC_i+2_, where i = 1…N) before applying the statistical functions. We opted for a stride-based segmentation instead of the step-based segmentation because a stride corresponds to one period of gait and, therefore, is more likely to capture the complete pattern of a cycle. Besides, the list of selected features (Table 1) aimed to collect information in the intensity of the signal (e.g., mean, STD, RMS), the shape of its distribution (e.g., skewness, kurtosis) and its shape in a compressed format (e.g., coefficient of the auto-regressive model). Moreover, as the temporal parameters (Equations 1–5) already hold relevant periodic information, we did not consider features in the frequency domain.

**Table 1:** List of the features extracted for each stride on the continuous acceleration *a(t)*, angular velocity ω*(t)*, speed *v*_*foot*_*(t)*, and slope *s(t)*.

**Type**	**Feature**	**Description**
Intensity	mean_ <*T*>_ <*C>*	Mean value
	std_ <*T*>_ <*C>*	Standard deviation
	med_ <*T*>_ <*C>*	Median
	iqr_ <*T*>_ <*C>*	Interquartile range
	max_ <*T*>_ <*C>*	Maximum
	rms_ <*T*>_ <*C>*	Root-mean-square
Shape	kurt_ <*T*>_ <*C>*	Kurtosis
	skew_ <*T*>_ <*C>*	Skewness
Compression	arm1_ <*T*>_ <*C>*	First coefficient of the auto-regressive model of order 3
	arm2_ <*T*>_ <*C>*	Second coefficient of the auto-regressive model of order 3
	arm3_ <*T*>_ <*C>*	Third coefficient of the auto-regressive model of order 3

*In the name of the feature, variables <T> and <C> correspond to the label of the signal and the channel, respectively. Hence <T> must be replaced by a, ω, v_foot_, or s while <C> must be replaced by x, y, z, or norm*.

Before proceeding to the selection of the best features, we visualized the relation between the reference speed *v*_*ref*_*(t)* and the features individually. Based on our observations, we identified three functions that improved the linear relationship between the reference speed and some of the input features; *f*_1_*(p)* = *p*^2^, *f*_2_*(p)* = *p*^3^, and *f*_3_*(p)* = *1/p*. The functions *f*_1_, *f*_2_, and *f*_3_ were applied to all the features, and the results added to the list of features. Finally, we also included several anthropometric parameters to the collection of features, such as the size, weight, gender, and age of the participants.

#### Data Set Configuration

**Figure 4:**
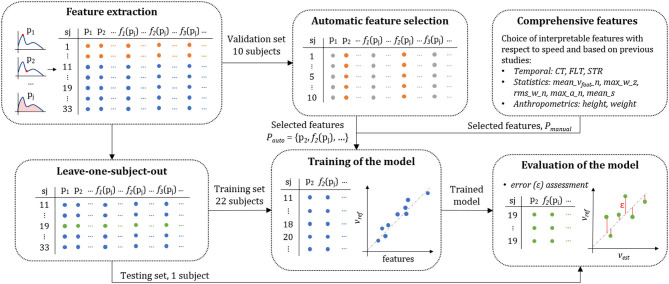
Schematic representation of the data repartition into the validation (blue), training (orange), and testing (green) set. The validation set was used for feature selection, the training set to train the coefficients of the linear model, and the testing set to evaluate the performance of the predictions. The features are represented as p_j_ and the linearization function as *f*_1_(p_j_), *f*_2_(p_j_), and *f*_3_(p_j_).

We divided the data into three subsets: validation, training, and testing sets. The participants were randomly distributed into the three subsets. It is important to note that all the steps of a single individual were attributed to only one of the subsets; this removed the performance bias associated with the models trained and tested on measurements originating from the same subjects (Halilaj et al., 2018). Figure 4 shows the data from each set with different colors and illustrates their functions.

We used the 10 subjects (30%) from the validation set for feature selection (in orange in Figure 4), and the 23 remaining participants (70%) were used interchangeably for training (in blue in Figure 4) and testing (in green in Figure 4) of the model according to the leave-one-subject-out cross-validation method. We emphasize on the fact that the validation set was not included in the evaluation of the model and served exclusively for feature selection. We distinguished the development set from the other sets to lessen the risks of overfitting and preferred a leave-one-subject-out approach for the assessment of the model's performance due to the relatively low number of individuals present in this study. Moreover, such a method allowed us to identify potential outliers in the participants and later find collections of subjects with similar biases.

The leave-one-subject-out cross-validation method functioned as followed: we trained the model using the data from 22 subjects (training set) and tested on the data from one individual (testing set). We then repeated this process, such that each participant appeared once in the testing set.

#### Automatic Feature Selection

Here, we selected the features (*P*_*auto*_) to minimize the mean-square error (MSE) of the speed estimation model using the ordinary least squares method. The leave-one-subject-out method was applied with 11 subjects for training and one subject to evaluate the error of the predictions (Figure 4). The automatic feature selection process started with an empty set of inputs and sequentially added the parameters p_j_ or their transform (*f*_1_, *f*_2_, *f*_3_), which minimized the average MSE among all the subjects. This method is known as the forward stepwise selection process and has proven to be reliable on large feature space (John et al., 1994; Kohavi and John, 1997). The algorithm stopped including new parameters if the gain in the average MSE was lower than 1% of the previous MSE recorded. We deliberately set a low 1% criterion to obtain a possibly unnecessary large number of inputs knowing that the model is trained using the LASSO method (Tibshirani, 1996) with shrinkage of the redundant features. To ensure that the features contributed equally to the MSE estimation, we rescaled the inputs using a robust z-score normalization method (Jain et al., 2005); after normalization, the feature's mean was equal to zero, and median absolute deviation equal to one (less sensitive to outliers than the variance of one).

#### Comprehensive Selected Features

Although a supervised and automatic feature selection method may retrieve the subset with the best prediction performance on a given set of parameters, the results are sometimes difficult to interpret. Hence it is generally recommended also to evaluate the performance of a comprehensive set of features selected based on their biomechanical relevance (Halilaj et al., 2018). Based on the findings of previous research in running, we defined a list of features (*P*_*manual*_) known to be affected by variations in the running speed. As for the automatic selection of features, we willingly selected a large number of input features, potentially intercorrelated, knowing that optional inputs will be discarded later in the training stage. In summary, comprehensive features included the following:

– Anthropometric features: the height because taller individuals are likely to have longer step length, thus higher speed, than shorter individuals with similar flight times.
– Temporal features: the CT, FLT, and STR contain relevant information about the stride frequency and were shown to decrease with an increase in the running speed (Saito et al., 1974; Nummela et al., 2007; Chapman et al., 2012).
– Speed and spatial features: the average speed of the foot (*mean_v*_*foot*_*_norm*) obtained with a direct integration; the maximum angular velocity of the foot in the sagittal plane (*max_*ω*_z*) assuming faster swing involves higher speed; the RMS value of the angular velocity norm (*rms_*ω*_norm*) since higher speed should result in higher dynamic movements; the maximum of the acceleration norm (*max_a_norm*) as it was demonstrated in previous studies that tibial peak accelerations increased with faster-running velocities (Sheerin et al., 2019); and the average slope (*mean_s*) since uphill and downhill may affect the running speed.

#### Training and Testing of the Model

The linear model was trained and tested with the leave-one-subject-out cross-validation method. For each individual, the performance of the speed prediction was evaluated with the model's coefficients trained on 22 other subjects. This approach was preferred to a traditional split of the data into two datasets (e.g., 70% training and 30% testing repartition) due to the restricted number of subjects available after the feature selection phase. Besides, the leave-one-subject-out procedure allowed us to detect potential outliers in the participants and, therefore, possibly identify the sources of poor estimation results.

The least-squares regression coefficients were trained using the LASSO method (Tibshirani, 1996), with scaled inputs to have zero mean and a variance of one, and equally distributed the observations' weights at the initialization stage. To limit the risks of overfitting, we selected the model with the smallest number of inputs, if any new input would improve the MSE by <2%.

Since we observed some disparity in the dataset (the steps between 2.5 and 4 m/s were over-represented), we used a random under-sampling (RUS) method to deal with the issue of class imbalance (Pes, 2020). This process started by dividing the range of reference speeds into five equally spaced groups, from 1.4 to 2.2 m/s, 2.2 to 3 m/s, 3 to 3.8 m/s, 3.8 to 4.6 m/s, and 4.6 to 5.4 m/s. We then randomly selected the same number of steps from each group based on the group with the least number of steps (i.e., down-sampling of the majority). We repeated this process ten times, generating ten versions of the under-sampled data set and used these subsets independently. In other words, we trained and tested the model 10 times for each individual.

Finally, we investigated the changes in the speed prediction when input features were averaged over consecutive steps. Instead of using a single step granularity for running speed, averaging over several steps might conceivably improve the precision (i.e., random error) of the model. We tested this approach on an even number of steps (i.e., 2, 4, 6, 8, and 10), for it equally includes the sensor's information from both feet. In order to avoid grouping non-consecutive steps, we applied this averaging process before under-sampling the inputs.

### Personalized Model

#### Running Speed Estimation Algorithm

Recently, online personalization methods have emerged in the field of human movement analysis. For instance, such an approach demonstrated significant improvement in speed estimation performances (Soltani et al., 2019). The objective is to personalize a generic speed estimation model based on the sporadic reference data obtained from a GNSS device. We describe the online-learning procedure used in this study in the following; we define *n* as the observation (or sample) index used for the personalization where each sample corresponds to one stride. Therefore, if we have M samples (i.e., strides) for the personalization, then *n* ∈ {1, 2, 3, …, *M* }.

Let's *Q* be the number features in each stride. We defined *p*_*n*_ as the feature vector and *sl*_*n*_ as the reference stride length for the *n*-th stride according to Equations (6, 7). Here, *p*_*j*_*[n]* is a symbolic name for the j-th feature of the n-th stride. Moreover, *v*_*ref*_[*n*] is the GNSS speed of the *n*-th stride.

(6) $$p_{n}=\left[\begin{array}{c} 1\;p_{1}[n]\;\begin{array}{c} p_{2}[n]\ldots \;p_{Q}[n] \end{array} \end{array}\right]$$

(7) $$sl_{n}=v_{ref}[n]\times \frac{1}{STR_{n}}$$

For *p*_*n*_ we used the selected features in *P*_*manual*_ or *P*_*auto*_ based on results obtained in the linear model. We first modeled the stride length through Recursive Least Square (RLS) and then multiplied that by the stride frequency to obtain the running speed. The RLS is a real-time and computationally effective online learning method, which does not need to have or store all the training data from the beginning of training.

Let *P*_*n*_ and *SL*_*n*_ be the feature matrix and the vector of actual stride length defined in Equations (8, 9), respectively.

(8) $$P_{n}=\left[\begin{array}{c} p_{1}\\ \vdots \\ p_{n} \end{array}\right]$$

(9) $$SL_{n}=\left[\begin{array}{c} sl_{1}\\ \vdots \\ sl_{n} \end{array}\right]$$

Using the RLS approach, *SL*_*n*_ can be modeled as in Equation 10, where β_*n*_ is the coefficient of the model trained using *n* observations. If *P*_*n*−1_ and β_*n*−1_ are the feature matrix and model coefficients estimated using *n-1* samples, then once we obtain a new sample (*p*_*n*_ and *sl*_*n*_) for the personalization, β_*n*_ can be recursively estimated through Equation (10).

(10) $$\beta_{n}=\beta_{n-1}+D_{n}p_{n}\left(sl_{n}-p_{n}^{T}\beta_{n-1}\right)$$

Where *D*_*n*_, known as the dispersion matrix, itself, is recursively estimated by having only *D*_*n*−1_ (i.e., the dispersion matrix estimated using *n-1* samples) and the new personalization data (i.e. *p*_*n*_ and *sl*_*n*_) according to Equation (11). Here, *K*_*n*_ is defined as Equation (12).

(11) $$D_{n}=D_{n-1}\left(I-p_{n}{\left(I+K_{n}\right)}^{-1}p_{n}^{T}D_{n-1}\right)$$

(12) $$K_{n}=p_{n}^{T}D_{n-1}p_{n}$$

For each individual, ten strides from the training set were used to initialize the recursion process of the RLS.

#### Cross-Validation

The data set was organized differently for the personalization process to consider the gait style of each individual and minimize the training data from GNSS. Data from each individual was divided into bouts of 10 strides, and half of these bouts were assigned randomly to the training set and the other half to the testing set of that same individual. Consequently, we trained and evaluated the models for each individual separately, using the uniquely the data from that same individual.

### Statistical Analysis

We evaluated the performance of the model by computing the error on the training and testing sets. We did so going from a single step to a ten-steps resolution according to the configuration of the inputs. For each of the RUS iteration, the intra-subject accuracy (or bias) and precision were estimated using the mean and standard deviation, respectively. The normality of the speed error was tested using the Lilliefors test, and in the case of non-normal distribution, the mean was replaced by the median and standard deviation by the Inter-Quartile Range (IQR). To better understand the performance of the system, the intra-subject RMS error was calculated, and the Pearson correlation coefficient was used to assess the linear dependence of the predictions. Since we used the leave-one-subject-out method for training and testing, the results were reported by computing the mean, the standard deviation, the minimum and the maximum on the intra-subject biases, precision, RMS error, and correlation coefficients. Agreement between the reference GNSS speed and the estimated speed was illustrated with Bland & Altman plots (Bland and Altman, 1986). Furthermore, to evaluate the distribution of the errors and possible overfitting, we used the cumulative distribution function (CDF) of step absolute error for both training and testing sets.

## Results

### Direct Speed Estimation

**Figure 5:**
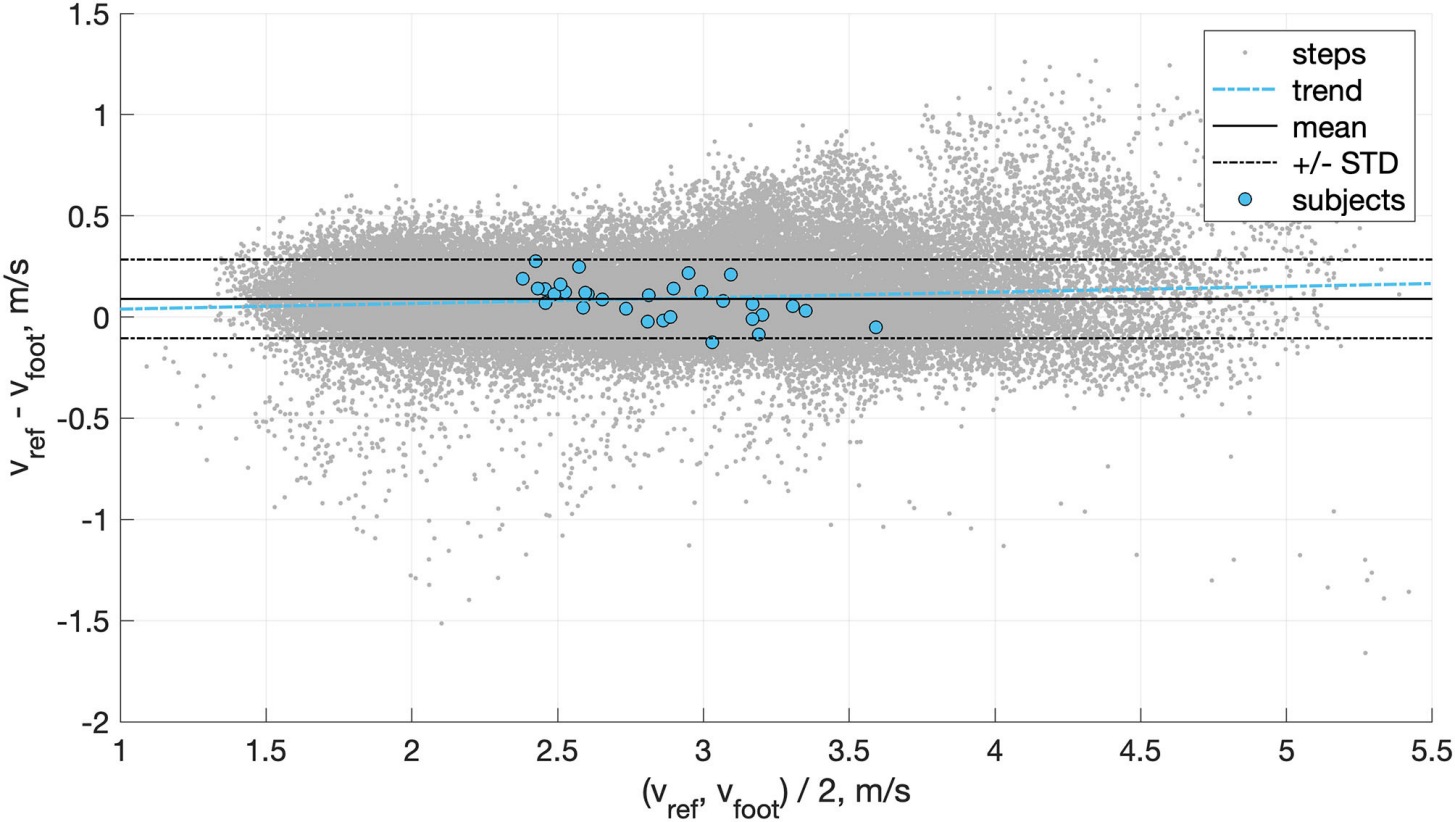
Bland-Altman plot of the agreement between the direct speed estimation method (*v*_*foot*_) and the GNSS reference (*v*_*ref*_). The error was estimated with a granularity of one step.

**Figure 6:**
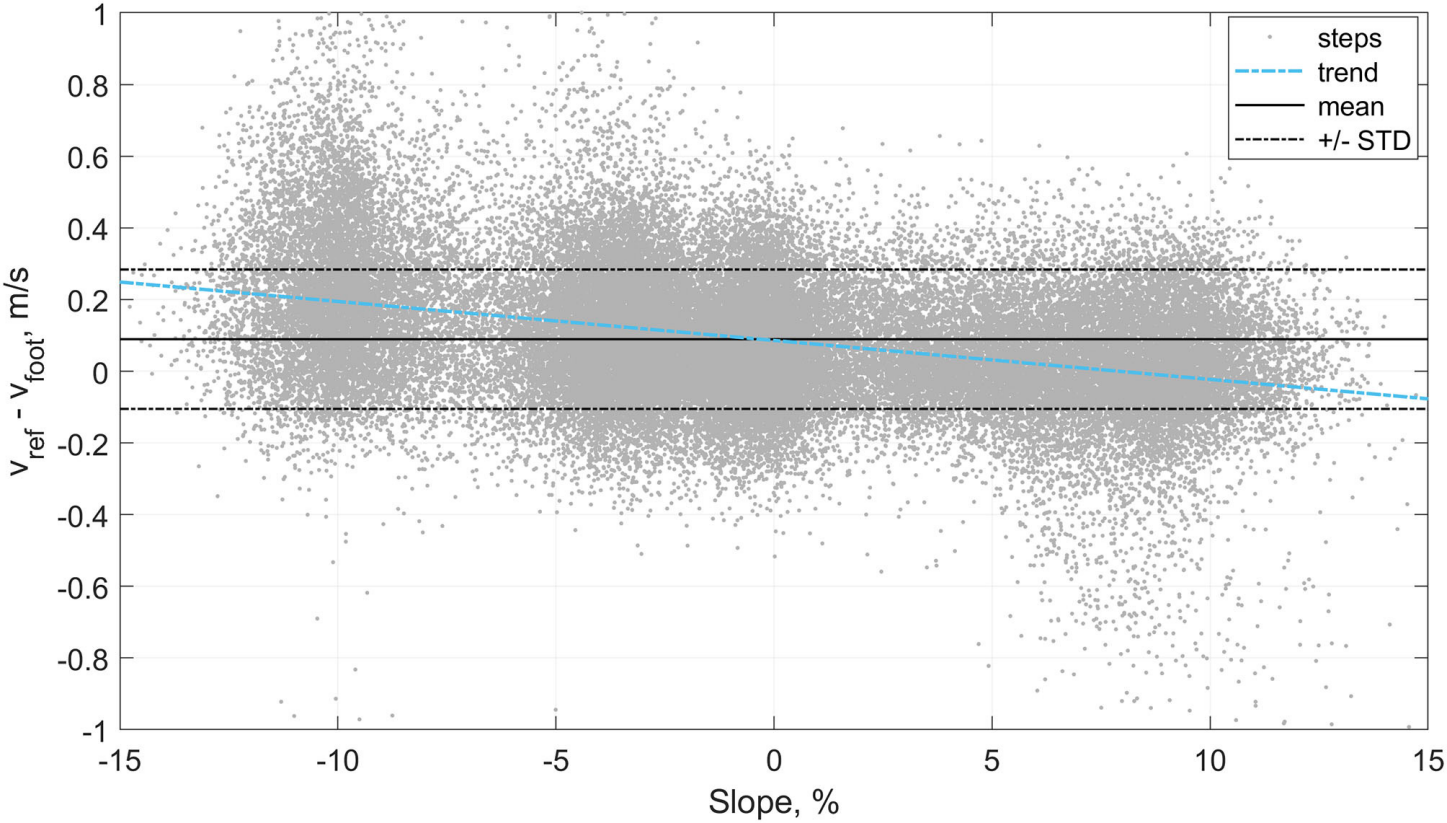
The step by step error of the direct speed estimation method (*v*_*foot*_) in relation to the slope of the ground surface.

Two subjects were excluded from the data set; because of the poor quality of the GNSS measurements or because of an improper fixation of the IMU on the shoe and high Signal to Noise Ratio (SNR) of the kinematic data. Since it required no learning, the direct speed estimation method was performed on the 63'435 steps available in this study. We observed an inter-subject mean ± STD (min, max) of 0.08 ± 0.10 (−0.12, 0.27) m/s for the bias, 0.16 ± 0.04 (0.08, 0.25) m/s for the precision, 0.20 ± 0.06 (0.08, 0.34) for the RMSE. The relation between the speed estimation error and the overground velocity is presented in Figure 5, and the effect of the slope in Figure 6.

### Automatic Feature Selection

**Figure 7:**
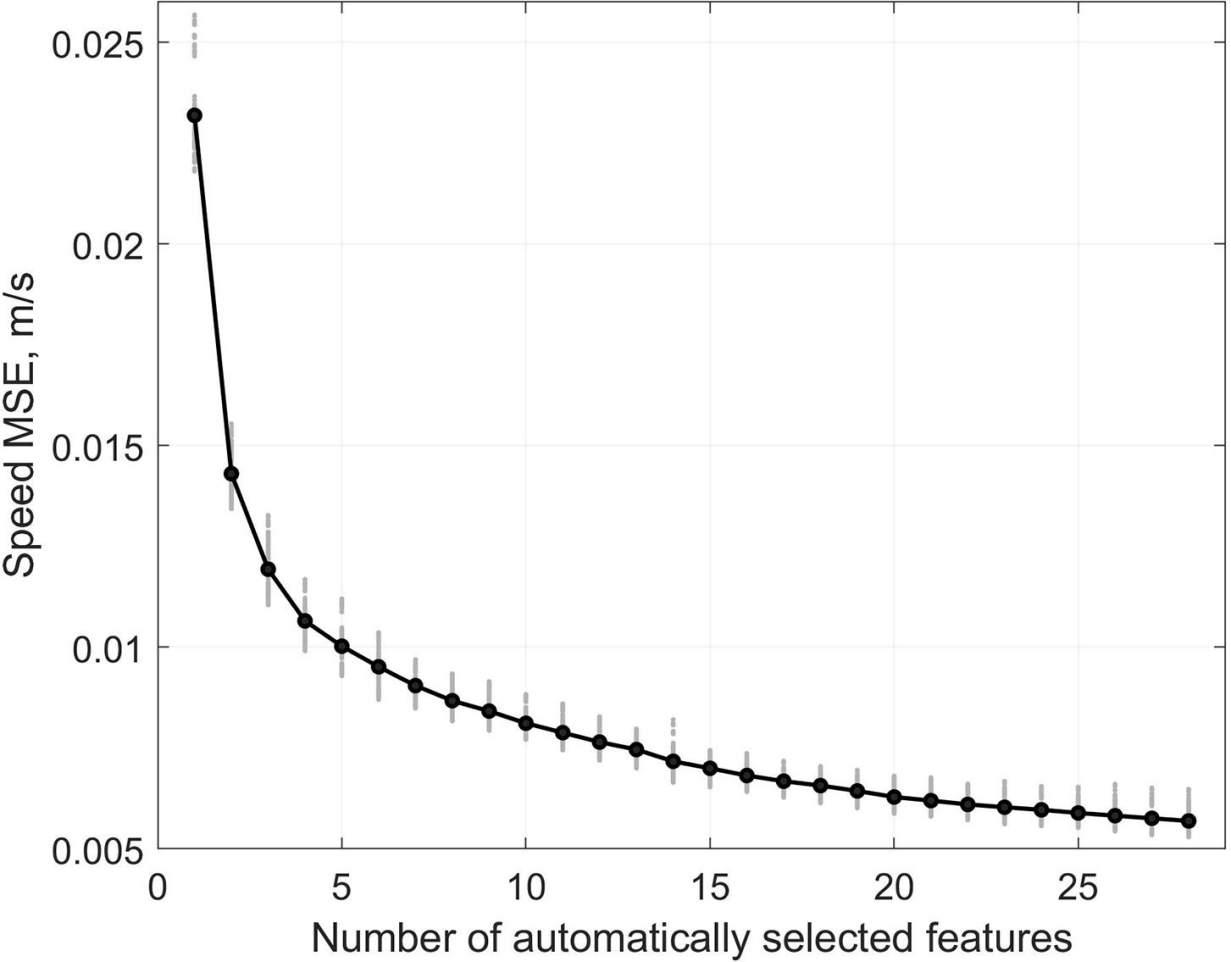
Mean Square Error (MSE) of the speed estimation during the forward stepwise selection process. In gray, the MSE of each subject and blue the inter-subject average.

In total, we used the 20'084 strides of the validation set to select 28 features out of the 668 features available. The feature selection process stopped at average Mean Square Error (MSE) of 0.0057 m/s (Figure 7), which corresponded to a 1.12% improvement compared to the previous step with 27 features. The selection process was repeated 100 times (i.e., 10 times for each of the 10 subjects) and led to the set of features presented in Table 2.

**Table 2:** The ordered list of the features automatically selected by the forward stepwise selection algorithm.

**#**	**Label**	***f(p)***	**#**	**Label**	***f(p)***
1	*mean_a_norm*	-	15	*mean_v_*foot*__y*	p^2^
2	*mean_v_*foot*__norm*	-	16	*median_ω_norm*	p^−1^
3	*iqr_a_norm*	-	17	*median_ω_x*	-
4	θ	-	18	*skew_v_*foot*__norm*	-
5	*mean_s*	p^2^	19	*iqr_v_*foot*__norm*	p^−1^
6	*STR*	p^−1^	20	*max_v_*foot*__y*	p^−1^
7	*median_a_x*	p^3^	21	*mean_ω_y*	-
8	*median_ω_z*	p^3^	22	*rms_a_x*	p^3^
9	*max_v_*foot*__norm*	-	23	*median_v_*foot*__x*	-
10	*median_a_y*	p^3^	24	*std_a_norm*	p^−1^
11	*mean_v_*foot*__x*	p^2^	25	*skew_ω_norm*	-
12	*skew_v_*foot*__y*	p^−1^	26	*skew_ω_z*	p^2^
13	*median_v_*foot*__y*	-	27	*std_a_x*	p^−1^
14	*std_ω_z*	-	28	*arm3_v_*foot*__y*	p^−1^

Out of the 28 features selected, 16 (57%) resulted from one of the three linearization functions (*f*_1_, *f*_2_, *f*_3_), one feature from the temporal analysis (STR), one from the orientation estimation (θ). The other features are statistics extracted from the different time series [i.e., acceleration *a(t)*, angular velocity ω*(t)*, the velocity of the foot segment *v*_*foot*_*(t)*, and the slope *s(t)*].

### Linear Model

In total, 43'351 steps were used to train and test the linear model. Due to the subdivision of the data associated with the leave-one-subject-out method, we used, for each individual, an average ± STD (min, max) of 41'287 ± 188 (41'032, 41'642) steps for training and 2'064 ± 188 (1'709, 2'319) steps for testing.

When the *P*_*auto*_ feature set was used for training, the LASSO method always favored the same 7 inputs (*P*_*auto,best*_) among the 28 features previously selected (Table 2):

$$\begin{array}{l} {\text{P}}_{\text{auto},\text{best}}=[\text{mean}\_\text{a}\_\text{norm},{\text{f}}_{1}(\text{mean}\_\text{s}),{\text{f}}_{3}(\text{STR}),{\text{f}}_{2}(\text{median}\_\omega \_\text{z}),\\ \text{ max}\_{\text{v}}_{\text{foot}}\_\text{norm},{\text{f}}_{1}(\text{mean}\_{\text{v}}_{\text{foot}}\_\text{y}),\\ {\text{ f}}_{3}(\text{median}\_\omega \_\text{norm})] \end{array}$$

In comparison, with *P*_*manual*_ the LASSO method selected 4 inputs (*P*_*manual,best*_):

$${\text{P}}_{\text{manual},\text{best}}=[\text{rms}\_\omega \_\text{norm},\text{mean}\_{\text{v}}_{\text{foot}}\_\text{norm},\text{mean}\_\text{s},\;\text{ CT}].$$

The performances of the linear predictor over the testing set are shown in Table 3; the inter-subject mean, STD, minimum, and maximum are presented for the bias, the precision, the RMSE, and the correlation coefficients. The results of the running speed estimation are presented for single-step resolution and also where the inputs were averaged over 2, 4, 6, 8, and 10 steps before being used by the linear model.

**Table 3:** Inter-subject mean, STD, minimum, and maximum of the system's bias, precision, Root-Mean-Square error (RMSE), and the linear correlation coefficient (R).

**Features**	**Steps**	**Bias (m/s)**				**Precision (m/s)**				**RMSE (m/s)**				**R**			
		**mean**	**STD**	**min**	**max**	**mean**	**STD**	**min**	**max**	**mean**	**STD**	**min**	**max**	**mean**	**STD**	**min**	**max**
*P_*auto,best*_*	1	0.00	0.10	−0.17	0.17	0.14	0.05	0.08	0.24	0.16	0.05	0.10	0.28	0.985	0.010	0.956	0.997
	2	0.00	0.11	−0.17	0.18	0.13	0.05	0.06	0.23	0.14	0.05	0.08	0.27	0.989	0.009	0.957	0.998
	4	0.00	0.11	−0.17	0.19	0.12	0.06	0.05	0.24	0.12	0.05	0.07	0.24	0.990	0.009	0.961	0.998
	6	0.00	0.11	−0.17	0.18	0.11	0.05	0.05	0.23	0.12	0.04	0.06	0.21	0.990	0.009	0.952	0.999
	8	0.00	0.11	−0.18	0.19	0.11	0.05	0.05	0.23	0.12	0.05	0.06	0.23	0.991	0.009	0.952	0.999
	10	0.00	0.11	−0.17	0.19	0.11	0.05	0.05	0.23	0.11	0.04	0.06	0.23	0.992	0.008	0.965	0.999
*P_*manual,best*_*	1	0.00	0.11	−0.22	0.17	0.15	0.06	0.09	0.29	0.18	0.07	0.11	0.37	0.983	0.009	0.961	0.997
	2	0.00	0.11	−0.23	0.18	0.13	0.06	0.07	0.26	0.15	0.06	0.09	0.29	0.988	0.008	0.963	0.997
	4	0.00	0.11	−0.23	0.20	0.12	0.06	0.06	0.26	0.14	0.06	0.08	0.24	0.989	0.009	0.959	0.998
	6	0.00	0.12	−0.23	0.19	0.12	0.06	0.06	0.24	0.13	0.06	0.06	0.24	0.990	0.009	0.956	0.999
	8	0.00	0.12	−0.23	0.20	0.11	0.06	0.05	0.24	0.13	0.06	0.06	0.24	0.991	0.009	0.944	0.999
	10	0.00	0.12	−0.24	0.20	0.11	0.06	0.05	0.24	0.12	0.06	0.06	0.24	0.991	0.008	0.964	0.999

*The results are presented for each configuration of inputs (*P*_*auto,best*_ and *P*_*manual,best*_)*.

**Figure 8:**
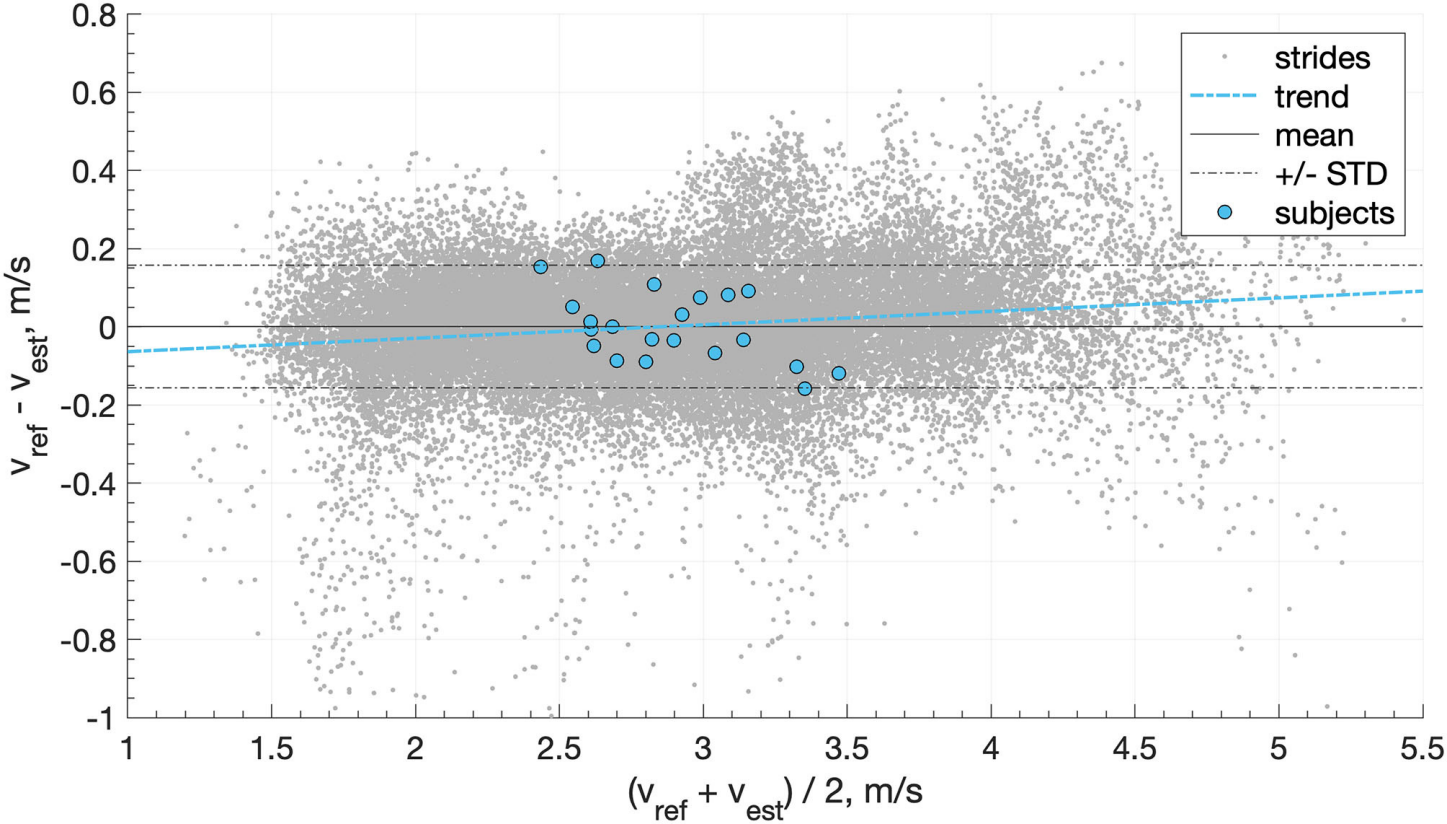
Bland-Altman plot of the speed estimation (*v*_*est*_) obtained with the features automatically selected (*P*_*auto,best*_) and compared with the reference GNSS speed (*v*_*ref*_). The gray dots represent the steps, the blue circle the average results of each subject, the solid black line the mean of the steps, the dashed black lines the STD of the steps, and the dashed blue line the linear trend of the steps.

In comparison, when we used a moving average (four steps) on the output of the speed estimation model (i.e., not the inputs as in Table 3), then we obtained an inter-subject mean ± STD (min, max) bias of 0.00 ± 0.10 (−0.17, 0.17) m/s, precision of 0.13 ± 0.05 (0.06, 0.23) m/s, RMSE of 0.14 ± 0.05 (0.08, 0.28) m/s, and correlation coefficients of 0.985 ± 0.010 (0.956, 0.997). The agreement between the speed estimation using *P*_*auto,best*_ (*v*_*est*_) and the reference GNSS system is presented for each stride (gray dots) and each individual (blue circles) in Figure 8.

**Figure 9:**
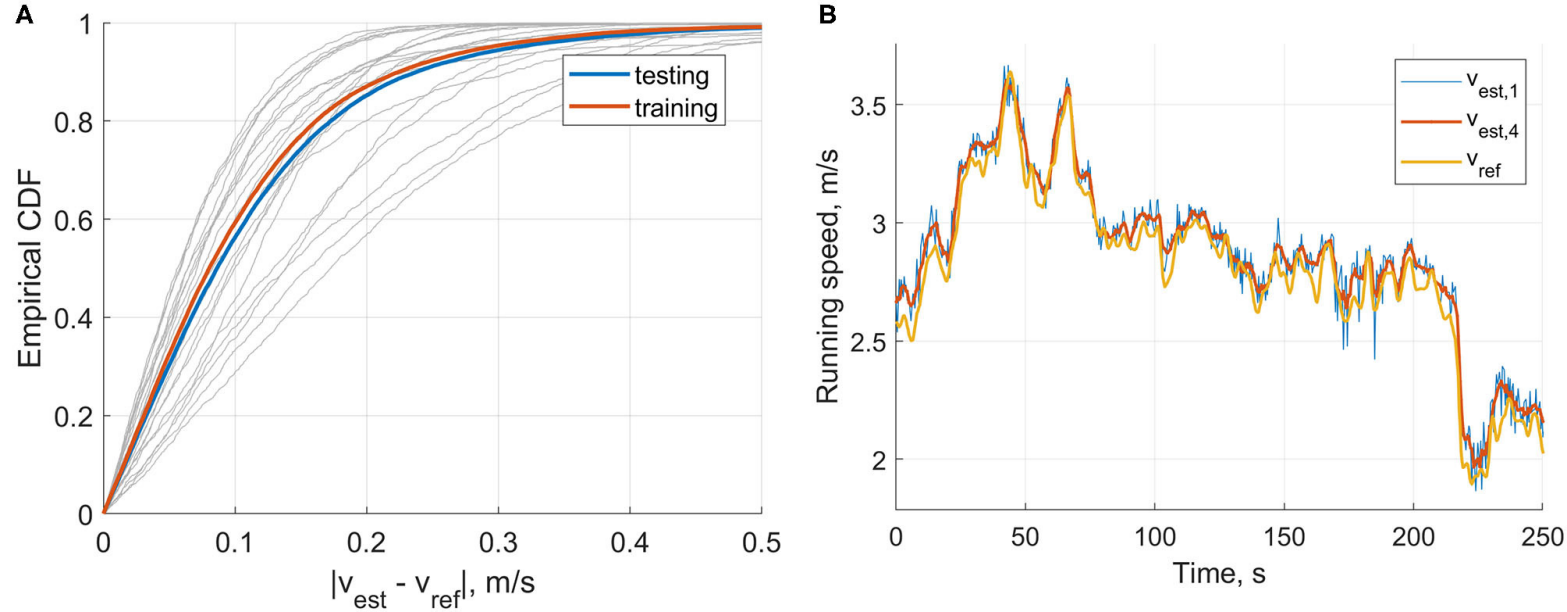
**(A)** The Cumulative Density Function (CDF) of the speed estimation error of each step (|*v*_*ref*_ – *v*_*est*_|). The speed was estimated using the automatically selected inputs (*P*_*auto,best*_). The gray curves represent the CDF of each individual in the testing set, the blue line the inter-subject CDF of the testing set, and the orange line the inter-subject CDF of the training set. **(B)** Comparison between the speed estimation of each step (*v*_*est*,1_), the speed estimation averaged over four steps (*v*_*est*,4_), and the reference GNSS speed (*v*_*ref*_).

Figure 9A shows the CDF of the speed estimation error for each subject (gray lines) and the subjects aggregated (blue line). In total, 56% of the recorded steps have an error below 0.1 m/s and 86% below 0.2 m/s. Finally, as illustration of overground measurement of speed over a various range of self-adjusted speed, the speed obtained with the reference GNSS system was compared for a typical subject with the speed estimation at step level (v_est_,1), and the estimation when averaged over four steps (v_est_,4) in Figure 9B.

### Personalization

**Figure 10:**
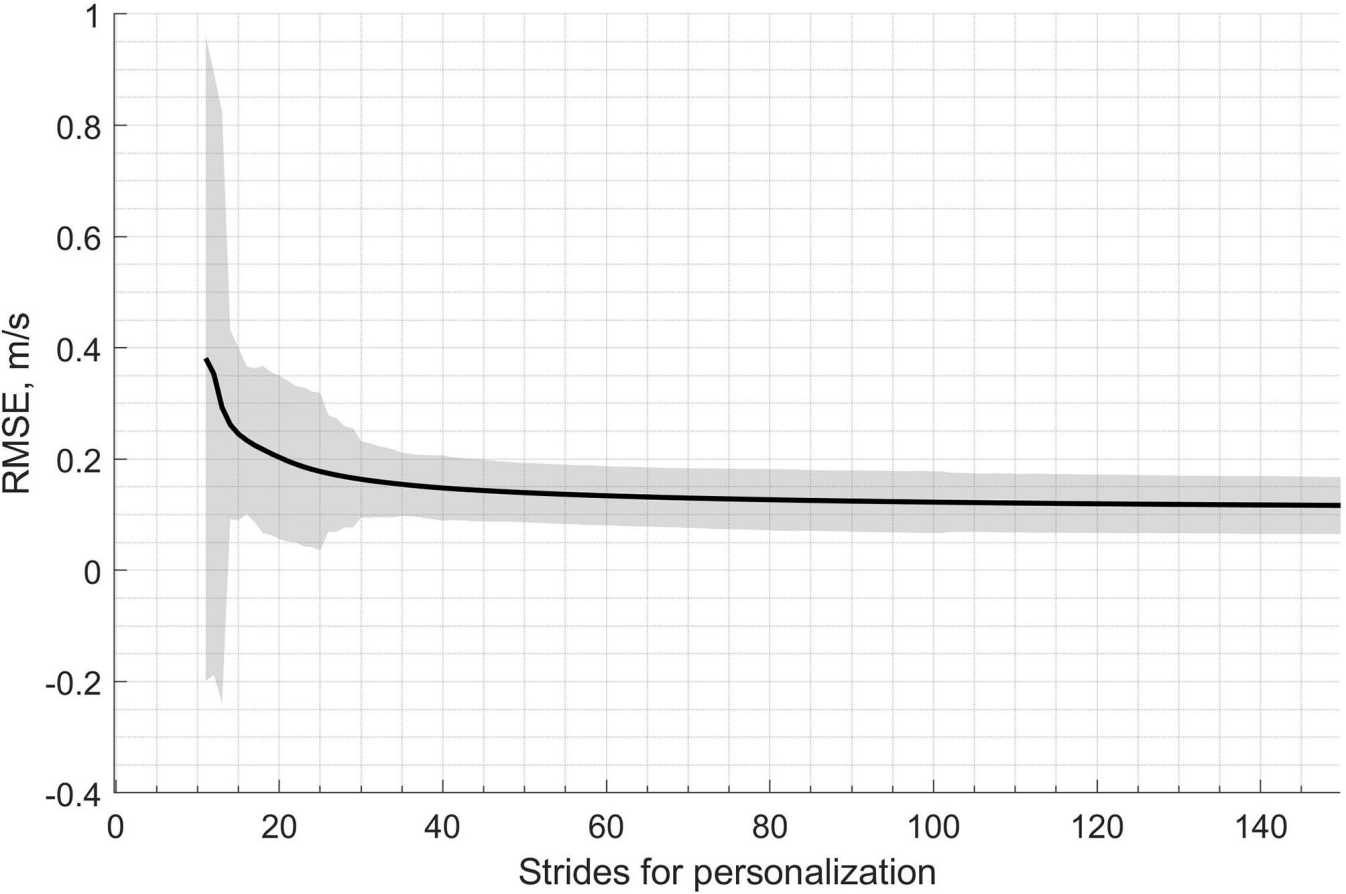
Evolution of the RMSE error during the personalization of the speed model. Here, the solid line and the shaded area represent, respectively, the inter-subject mean and standard deviation of the RMSE. The x-axis corresponds to the number of strides used for the personalization. Note that, for a better visualization of the error evolution, the figure is zoomed only on the first 150 samples used for personalization.

We used the features in *P*_*manual*_ to train and test the personalized model since the results of the generic model show little differences between *P*_*auto,best*_ and *P*_*manual,best*_, and because, with P_*manual*_, we could include the 10 subjects from the validation set in the training and testing process without any risk of overfitting. For each subject, the training samples (i.e., half of the data of the subject, randomly selected) were fed one-by-one to the RLS, and the speed was estimated with the complete test set of the subject. Figure 10 shows this process for the first 150 strides used for personalization of the model; the solid line and the shaded area represents the inter-subject mean and standard deviation of the RMSE, respectively. Also, the evaluation error for the first 10 strides is not displayed in Figure 10; these strides were used to initialize the RLS algorithm.

**Figure 11:**
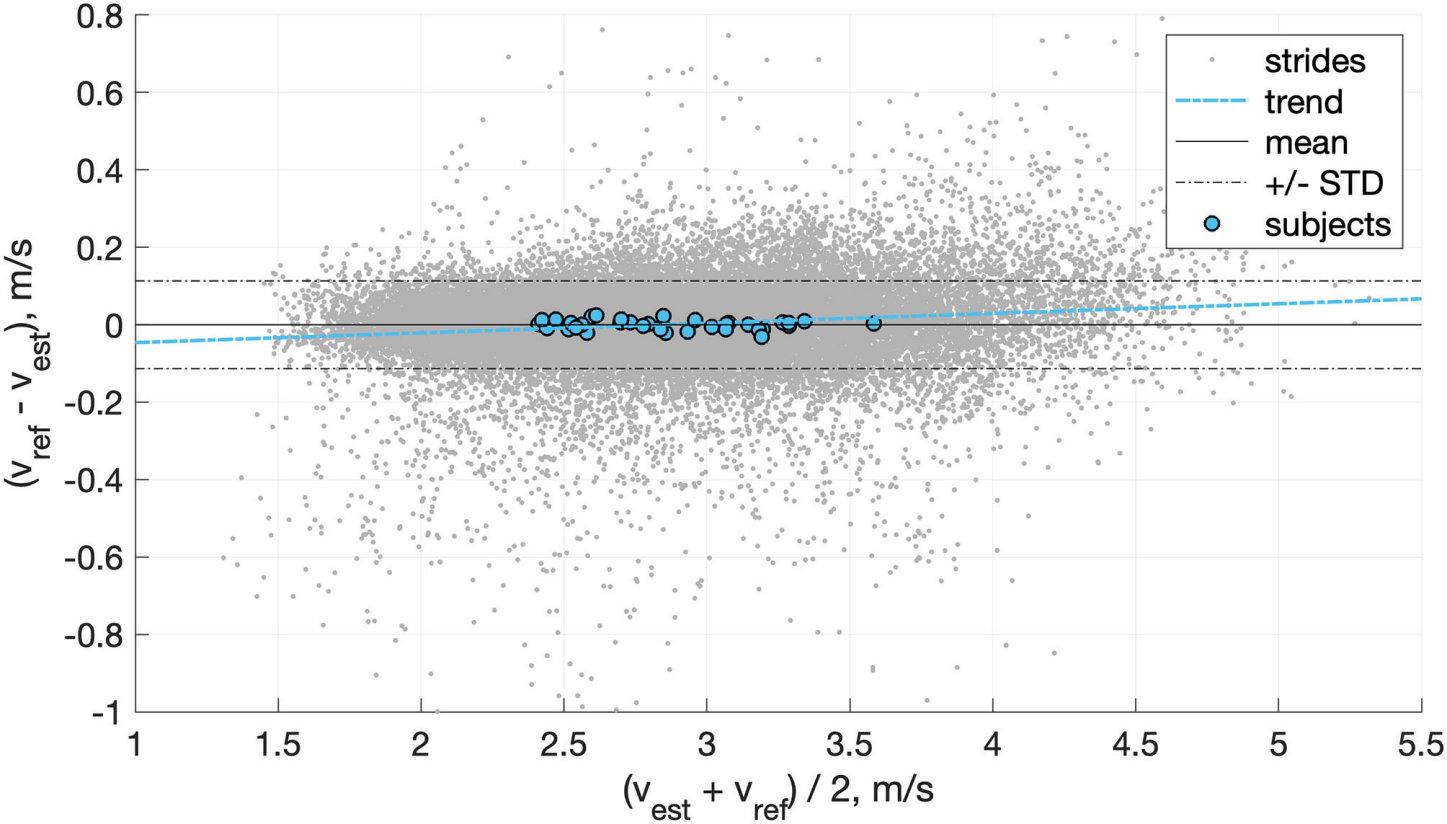
Bland-Altman plot of the proposed personalized model. Here, the points represent samples in the testing of all subjects. The dark and dotted and lines show a mean and standard deviation of the error, respectively.

In total, we used 1,139 ± 149 strides for training and 1,132 ± 149 strides for testing for each individual. Table 4 reports the bias, precision, and RMSE of the personalized model. Figure 11 also shows the Bland-Altman plot of the personalized model where the mean and standard deviation of the error is displayed by the dark and dotted lines, respectively. Moreover, the Spearman's test showed a high correlation of 0.97 between the estimated and the reference values of running speed.

**Table 4:** Inter–subject median and Inter-Quartile Range (IQR) of bias, precision, and RMSE of the personalized model.

**Bias (m/s)**		**Precision (m/s)**		**RMSE (m/s)**	
**median**	**IQR**	**median**	**IQR**	**median**	**IQR**
0.00	0.01	0.09	0.03	0.09	0.06

## Discussion

In this study, we proposed three methods to estimate overground running speed using feet worn sensors. First, we estimated the overground speed using solely the velocity of the foot obtained through the direct integration of the acceleration. We evaluated this direct method to test our hypothesis that the accelerometer fails to provide the correct value during the flight phase due to the combination of rotational and translational accelerations. Nevertheless, the velocity of the foot, with other relevant features, was selected as the input of the second method based on a linear model to predict the running speed. Thanks to an exhaustive features selection procedure and cross-validation approach, the model predicted the running speed with better accuracy. Finally, we assumed that the running technique varies among individuals, but that it should be well-correlated with individual gait features. Therefore, we showed that the performance of running speed could be improved using an online-personalization method with sporadic access to some GNSS data. It is important to note that the same method could be extended to less complicated instrumentation (e.g., a stopwatch over a fixed distance).

The speed estimation result for the method based on *v*_*foot*_ only confirmed our hypothesis that the direct integration of the acceleration, as proposed for walking, cannot be generalized to running due to the presence of aerial phases. The inter-individual mean bias (0.08 m/s) we observed indicates that the direct integration method underestimates the speed during the phase of flight. This underestimation confirms the inexact measure of the translational movement by the accelerometer during the flight phase. Moreover, the trend displayed in the Bland-Altmann plot (Figure 5) indicates that the system underestimates the velocity more at faster speeds. This observation is coherent with our hypothesis; the higher the speed, the greater the distance covered during the phase of flight (i.e., longer step length) (Nummela et al., 2007). Slope also seems to be a confounding factor of the error (Figure 6), with higher errors obtained during downhill running. In conclusion, *v*_*foot*_ itself does not characterize the speed of the subject as it cannot measure the distance covered during the period of the flight, but *v*_*foot*_ was a good proxy for speed and was one of the main features for speed prediction based on the linear model.

The selection of relevant features in the linear model was a crucial phase. Feature selection was carried over 20'084 steps and aimed to retrieve the most relevant features among the 668 variables available. Although we used a high-dimensional feature space, the curse of dimensionality issue did not apply as we used approximately 30 times more observations for feature selection. The results of the feature selection process show that the cost function (i.e., MSE) decreased quickly with the first few inputs and then stabilized as additional features were included (Figure 7). We set the stopping criteria intentionally low (i.e., 1% improvement in the MSE), knowing that the LASSO method used for training the model would ignore the inputs with redundant information. Interestingly, several of the features manually selected (*P*_*manual*_) were among the first to be selected by the automatic process (*P*_*auto*_); however, using different linearization functions (Table 2).

The linear model required inputs parameters from the temporal and spatial domain, as well as overground slopes. Hence a precise estimation of related parameters is paramount to optimize the precision of the speed estimation. The methods used to obtain these parameters should always be carefully reported and, ideally, previously validated. Interestingly, the model did not select the FLY parameter and instead favored the inverse of the stride duration (i.e., the stride frequency); hence none of the features selected required a bipedal configuration of the sensors allowing us to use the model with a single foot-worn IMU in the future. Also, none of the anthropometric parameters was necessary for the estimation of the running speed. This result is somewhat surprising, as we expected the height to be an essential input.

Apart from its computation time greediness, one reported issue of the forward selection algorithm is that decisions made early in the process cannot be changed, therefore potentially affecting its performance when the inputs are correlated (Derksen and Keselman, 1992). Although we observed some correlation in the inputs, we presumed that the two-fold selection process (i.e., stepwise selection and LASSO) would not be significantly affected by that matter. Moreover, the linearization of the feature-space was an essential component of this study. We selected *f*_1_, *f*_2_, and *f*_3_ functions based on visual inspection of the data, and out of the 28 pre-selected features, 16 (57%) resulted from these linearization functions.

Although the performance of the automatically selected set of features (*P*_*auto,best*_) performed slightly better than the comprehensive set of features (*P*_*manual,best*_), the differences remain in the order of a few centimeters per second (Table 3). Indeed, the estimations based on *P*_*auto,best*_, with a granularity of 1, over-performed the ones using *P*_*manual,best*_ by 0.01 m/s in the inter-subjects STD of the bias, 0.01 m/s in average precision, and display a slightly lower RMSE. These differences are relatively little since several elements in *P*_*manual,best*_ were among the most relevant features selected by the LASSO regression method in *P*_*auto,best*_, or at least were highly correlated. The results also show that averaging the inputs over several steps had a moderate effect on the performance of the system; it reduced the random error of the system with mean precision values consistently decreasing from 0.14 m/s for the step level estimation to 0.11 m/s when the granularity decreased to 10 steps. Also, when the output of step level estimated speed was averaged over four steps, the precision slightly improved (0.13 ± 0.05 m/s). Hence, whether the inputs or the outputs are averaged does not seem to affect the performances of the model.

Overall, the linear method showed good prediction results across a wide range of speed and slope, observed in real-world conditions (Figure 9B). It principally removed the mean bias of the method based on *v*_*foot*_ only and slightly improved the precision. The Bland-Altmann plot in Figure 8 shows a good agreement between the linear model and the reference GNSS system. The linear trend of the error (dashed blue line) is almost horizontal (y = 0.0034x+0.098), which suggests that the running speed has little effect on the error. These results support the usage of the RUS technique on the training data; the model ensured that all the ranges of speeds observed were equally represented. Although procedures more sophisticated than the RUS method have been proposed, they do not always provide a clear advantage in the results (Japkowicz, 2000). Moreover, the CDF curves of the training and testing sets do not indicate clear overfitting of the training data (Figure 9A) as the training set attains better performance than the testing set, but these are within an acceptable range.

It seems challenging to reduce further the STD of the bias using such a linear model since it depends on the inter-subject differences as it has previously been reported that individuals use different spatiotemporal adaptations at similar speeds. For instance, previous studies have shown that the relationship between stride frequency and stride length was specific to each subject (Saito et al., 1974; Nummela et al., 2007). These limitations were also encountered by previous studies that aimed to estimate the running speed based on body-worn inertial sensors. In Yang et al. (2011), the authors used a shank-worn IMU to measure the velocity of the shank and compared it with the speed of a motored treadmill. The study was conducted at five predefined speeds (2.5, 2.75, 3, 3.25, 3.54 m/s), with seven participants, and the error was calculated as the difference between the average estimated speed over 30 strides and the constant speed of the treadmill (i.e., the bias). The results show inter-trial mean and STD of the bias of 0.11 ± 0.03 m/s at 2.5 m/s, 0.10 ± 0.03 m/s at 2.75 m/s, 0.08 ± 0.02 m/s at 3 and 3.25 m/s, and 0.09 ± 0.02 at 3.5 m/s. The biases reported in Yang et al. (2011) are in range with those obtained in our study. However, the measurements were performed on a leveled treadmill at a discrete and limited number of running speeds, and the results were averaged over 30 strides (i.e., 60 steps). By considering the foot and shank as a single rigid body, the authors in Chew et al. (2017) used foot-worn inertial sensors with ten participants and a similar approach as in Yang et al. (2011). Based on the errors reported at each speed (8, 9, 10, 11 km/h), our method outperformed the one proposed in Chew et al. (2017). Aiming to evaluate the accuracy and the repeatability of a commercialized foot-worn running assessment system (RS800sd, Polar, Kempele, Finland), the authors in Hausswirth et al. (2009) performed 30-s measurements at multiple speeds (from 12 to 18 km/h) and compared the speed estimations with the speed of the treadmill. Even though the commercialized system required a subject-specific calibration, the reported mean ± STD bias of −0.03 ± 0.14 m/s indicates a slightly less accurate estimation of the running speed than the method proposed in this study. In a study (Herren et al., 1999) conducted in outdoor conditions, the authors explored whether triaxial accelerometric measurements can be combined with subject-specific neural networks to assess speed and incline of running accurately. The authors reported an RMSE of 0.12 m/s for average speed the whole running trial which is similar to our linear model estimations when the inputs are averaged at least four steps.

In a recent effort to reduce the inter-subject differences in the bias, researchers in De Ruiter et al. (2016) proposed a personalized speed estimation model based solely on the measurement of the contact time (CT). They obtained the CT using shoe-worn inertial sensors and conducted the measurements on an outdoor 2 km long tarmac. First, they personalized a model (*speed* = αCT^d^) for each of the 14 participants based on the average speed over several bouts of 125 meters. Then, they compared the personalized estimation results with those obtained with a stopwatch over a fixed 120-meters distance (*N* = 35 bouts) and reported a median RMSE of 2.9 and 2.1% (two runs). In comparison, our linear model method obtained a mean RMSE of 5.1% at step level estimation, and the personalized method a median RMSE of 3.1 %. This slightly higher RMSE in our study is partly reflecting the variety of slopes in our measurements in comparison to the level running in De Ruiter et al. (2016).

A recent study (Soltani et al., 2019) proposed a real-world speed estimation method based on wrist-worn inertial sensors. The authors obtained a median [IQR] (Inter-Quantile Range) bias of −0.02 [−0.2, 0.18] m/s and precision of 0.31 [0.26, 0.39] m/s for the non-personalized method. These results improved using a personalization technique similar to this study, with 0.00 [−0.01, 0.02] and 0.18 [0.14, 0.23] for the bias and precision, respectively. Hence, for both the personalized and non-personalized methods, this study out-performed the wrist-based estimation of the running speed.

The linear model is accurate for “average people” (i.e., individuals with typical running patterns), and individuals with an atypical running technique will give rise to higher speed estimation errors (Figure 8). In comparison, the personalized model adapts to the movements of each individual; thus, it ensures a bounded error for “average” and “atypical” individuals (Figure 11).

The proposed personalization demonstrates significant improvements in the performance of the real-world running speed estimation. As reported in Table 4, the personalization process improved the IQR of the bias by at least a factor of 10 and the median precision by roughly 30% by employing approximately 35 times less training data than the non-personalized linear model. The personalized model bypasses the bias caused by the intrinsic variation of individuals during real-world running. This observation is best characterized by Figure 10, which demonstrates the relatively fast convergence of the proposed RLS-based personalization; after roughly 50 strides, the model stabilized. As a consequence, the personalized model does not require continuous GNSS value to be updated. Once a good performance is reached, GNSS switch to off to save batteries. Moreover, the proposed personalized method is based on an online learning technique that does not require a database; hence it saves time and energy. It allows real-time speed estimation, computationally optimized, and does not need to store training data.

## Conclusion

In this study, we proposed and evaluated three different methods for real-world speed estimation in running: direct speed estimation, training based linear model, and a personalized model. The direct estimation of the foot velocity confirmed the hypothesis that accelerometers inaccurately measure the translational motion of an individual during the flight phase; therefore, techniques developed for walking analysis cannot be generalized to running. We evaluated the linear model for two sets of features: automatically selected (i.e., optimized) or manually selected (i.e., comprehensive features). The model performed best when we averaged its output over a few steps and showed that 4 steps (i.e., two left strides and two right strides) provided an acceptable trade-off between performance (bias: 0.00 ± 0.11 m/s; precision: 0.12 ± 0.06 m/s) and time-resolution. The personalized method tested in this study, used an online-learning technique based on recursive least-squares to personalize the speed estimations for each individual. Our results indicate that such an approach primarily helps to reduce the inter-subject bias (0.01 m/s) but also improves the average random error by more than 30%.

Based on the results of this study, we recommend using the linear model for speed estimation when the recordings of other accurate devices are temporarily unavailable and personalized the model when these recordings are available. For instance, the system can be used as a complement to a GNSS device experiencing sparse communication, either due to a reduced transmission bandwidth (e.g., indoor running, city centers) or because of electrical power limitations (e.g., low power systems).

## Data Availability Statement

The raw data supporting the conclusions of this article will be made available by the authors, without undue reservation.

## Ethics Statement

The studies involving human participants were reviewed and approved by EPFL's Human Research Ethics Committee. The patients/participants provided their written informed consent to participate in this study.

## Author Contributions

MF, AS, and KA conceptualized the study design and contributed to the analysis and interpretation of the data. AS conducted the data collection. MF and AS designed the algorithms and KA supervised the study. MF drafted the manuscript, all other authors revised it critically. All authors approved the final version, and agreed to be accountable for all aspects of this work.

## Conflict of Interest

The authors declare that the research was conducted in the absence of any commercial or financial relationships that could be construed as a potential conflict of interest.
